# Effectiveness of atrial fibrillation rotor ablation is dependent on conduction velocity: An *in-silico* 3-dimensional modeling study

**DOI:** 10.1371/journal.pone.0190398

**Published:** 2017-12-29

**Authors:** Byounghyun Lim, Minki Hwang, Jun-Seop Song, Ah-Jin Ryu, Boyoung Joung, Eun Bo Shim, Hyungon Ryu, Hui-Nam Pak

**Affiliations:** 1 Yonsei University Health System, Seoul, Republic of Korea; 2 Department of Mechanical and Biomedical Engineering, Kangwon National University, Chuncheon, Ganwon-do, Republic of Korea; 3 NVIDIA, Yonsei University, Department of Mathematics, Seoul, Republic of Korea; University of Minnesota, UNITED STATES

## Abstract

**Background:**

We previously reported that stable rotors are observed in *in-silico* human atrial fibrillation (AF) models, and are well represented by a dominant frequency (DF). In the current study, we hypothesized that the outcome of DF ablation is affected by conduction velocity (CV) conditions and examined this hypothesis using *in-silico* 3D-AF modeling.

**Methods:**

We integrated 3D CT images of left atrium obtained from 10 patients with persistent AF (80% male, 61.8±13.5 years old) into *in-silico* AF model. We compared AF maintenance durations (max 300s), spatiotemporal stabilities of DF, phase singularity (PS) number, life-span of PS, and AF termination or defragmentation rates after virtual DF ablation with 5 different CV conditions (0.2, 0.3, 0.4, 0.5, and 0.6m/s).

**Results:**

1. AF maintenance duration (p<0.001), spatiotemporal mean variance of DF (p<0.001), and the number of PS (p = 0.023) showed CV dependent bimodal patterns (highest at CV0.4m/s and lowest at CV0.6m/s) consistently. 2. After 10% highest DF ablation, AF defragmentation rates were the lowest at CV0.4m/s (37.8%), but highest at CV0.5 and 0.6m/s (all 100%, p<0.001). 3. In the episodes with AF termination or defragmentation followed by 10% highest DF ablation, baseline AF maintenance duration was shorter (p<0.001), spatiotemporal mean variance of DF was lower (p = 0.014), and the number of PS was lower (p = 0.004) than those with failed AF defragmentation after DF ablation.

**Conclusion:**

Virtual ablation of DF, which may indicate AF driver, was more likely to terminate or defragment AF with spatiotemporally stable DF, but not likely to do so in long-lasting and sustained AF conditions, depending on CV.

## Introduction

Atrial fibrillation (AF) is the most common sustained arrhythmia due to irregular electrical activity in the atrium, and is the major cardiac cause of stroke. About 1% of all adults have been diagnosed with AF, and the prevalence of AF is associated with increasing age, with a frequency of 9% in individuals 80 years old or older [1]. The rate of AF is expected to double by the year 2050 [1, 2]. Catheter ablation is an established standard treatment modality for anti-arrhythmic drug resistant AF. Although circumferential pulmonary vein (PV) isolation has been accepted as the cornerstone technique for AF catheter ablation [3], complex atrial substrates and non-PV triggers contribute to the initiation of AF and PV isolation alone does not achieve a satisfactory rhythm outcome especially in patients with persistent AF [4]. To overcome this technical limitation of AF catheter ablation, Narayan et al. mapped the rotor of AF using a multi-electrode basket catheter, and ablation of the rotor terminated or slowed AF and improved rhythm outcome [5]. We previously reported on AF rotors well identified by dominant frequency (DF) sites using both 2D and 3D human *in-silico* LA models [6], and effective AF termination or defragmentation of AF by virtual ablation targeting the highest DF areas [7, 8]. High DF areas may posit rotors or drivers of AF [9, 10], and ablation of high DF areas terminates or defragments AF [10]. There were several clinical studies reporting DF as an appropriate target for ablation, AF rotor, or driver [9–11]. In contrast, given the controversy surrounding this topic, leading circle and multiple wavelet theory [12] opposed the existence of rotor in AF and experimentally verified by double-layer hypothesis [13]. Li et al. reported that the spatiotemporal consistency of the DF area was observed in only 10% of AF episodes based on our modeling study [14]. Sequential electrogram acquisition may raise concerns about DF stability, and variations in the distribution of DF during long-lasting AF have been reported to be spatiotemporally unstable in the clinical field [15]. The RADAR-AF clinical trial failed to prove the superiority of DF source ablation outcomes compared to conventional ablation [16]. To explain the potential mechanisms for rotor ablation was successful in some clinical studies, but not in others, we hypothesized that the spatiotemporal stability of an AF rotor is variable depending on the conduction velocity (CV) which is one of major determinants for spatiotemporal stability of DF site or rotor and a proportional relationship with wavelength. Computer simulation model have an advantage since they allow tests to be repeated under various conditions from single cells to entire tissue regions precisely and reproducibly. The purposes of this study are to verify the spatiotemporal stability of DF, which is represented by a rotor, with 5 different CV conditions in 3D patient-specific LA models of AF, and to evaluate the outcomes of virtual ablation for high DF sites and its relationship with rotor stability.

## Methods

The study protocol was approved by the Institutional Review Board of Severance Cardiovascular Hospital, Yonsei University Health System, and adhered to the Declaration of Helsinki. This study is registered at Clinicaltrials.gov (NCT 02171364). All subjects provided written informed consent.

### 3D atrial cell and tissue model

The *in-silico* 3D model of the human left atrium (LA) was generated by the EnSite NavX system (Endocardial Solutions, St. Jude Medical, Inc., St. Paul, MN, USA) using computed tomographic (CT) image data from patients with of clinically persistent AF. After generating 3D geometry using the NavX system, a 3D mesh was generated and refined by the customized software (CUVIA, Model: SH01, ver. 1.0; Laonmed Inc., Seoul, Korea) with triangular type. Each triangular mesh consisted of three nodes, which are located at the vertices of each corner, and between 440,000 and 500,000 nodes were generated in the model. Mesh size, which is the distance between two adjacent nodes, was approximately 300 μm. In-silico model was homogeneous, and it was applied to a single cell by using the monodomain model. For 3D-simulation of electrical wave propagation in cells, the following monodomain equation Eq (1) was used [17, 18]:

$$\frac{\partial V_{m}}{\partial t}=D\nabla^{2}V-\frac{I_{ion}+I_{stim}}{C_{m}},$$ (1)

where V_m_ is the membrane potential, D is the conductivity tensor, I_ion_ and I_stim_ are the ion current density and stimulation current density, respectively, and C_m_ is the membrane capacitance per unit area. The AF model was performed using CUDA 6.5 in Microsoft Visual Studio 10.0 (Microsoft Co., Redmond, WA, USA). The ionic currents in each cell were determined using the human atrial myocyte model developed by Courtemanche et al. For the remodeling of ion currents of AF, I_to_, I_Kur_, and I_CaL_ were reduced by 80%, 50%, and 40%, respectively [19], and I_K1_ was increased by 50% [20]. The study included 10 patients with persistent AF (8 men, mean age 61.8±13.5 years old). We chose 5 different CVs of 0.2, 0.3, 0.4, 0.5, and 0.6 m/s, to investigate the spatiotemporal stability of DF at each CV [21, 22]. The CVs were chosen based on real human patient data (Yonsei AF ablation cohort data; n = 1,980; mean CV = 0.43 ± 0.24 m/s) [23]. Diffusion coefficient was adjusted for each CV, and proportionally referenced to previous studies (surface CV 0.72 m/s at 500 ms pacing cycle length under diffusion coefficient 0.00154 cm2/ms) [6, 24]. CVs were calculated from time and distance of wave propagation between high septum to tip of LA appendage. Finite element formulation was used for electric wave propagation on triangular mesh [25]. Time step was adaptively varied between 0.01 and 0.1 ms. Diffusion stability was set to satisfy the Courant-Friedrichs-Lewy condition. No flux condition was applied for all boundaries.

### AF initiation, DF and PS generation, and the analysis algorithm

**Fig 1:**
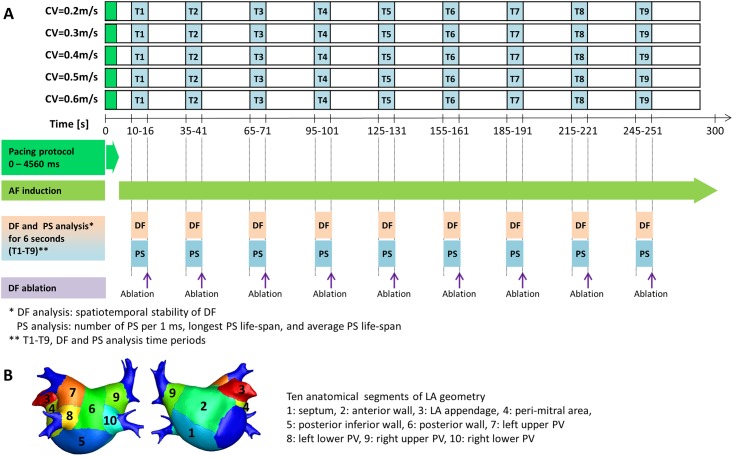
**A.** Study protocol. Ramp pacing stimulation was performed for 4,560 ms, and AF is observed after AF induction with 5 different CV conditions counting AF maintenance duration (max 300 s). DF analysis (spatiotemporal stability of DF) and PS analysis (number of PS per 1 ms and the longest PS life-span) were performed, and virtual DF ablations were targeted to the areas with the 10%, 15%, and 20% highest DF area at each CV conditions. **B.** Ten anatomical segments of the LA geometry.

A diagram of the AF induction protocol and analysis is shown in Fig 1A. Ramp pacing stimulation was applied at the high septum of the LA near Bachmann’s bundle insertion to induce AF. This resulted as Bachmann’s bundle is one of the main inter-atial conduction pathways from sinus node to left atrium, and we simulated conditions for AF induction in the clinical electrophysiology laboratory using high right atrial rapid pacing, according to our previous simulation studies [7, 14]. A total of 24 pacing stimuli were performed with cycle lengths of 200, 190, and 180 ms. The overall pacing duration was 4,560 ms. After induction of AF, we observed AF maintenance for 300 s with the 5 different CV conditions counting AF maintenance duration (max 300 s). We defined ‘successful AF induction’ as AF maintained for longer than 20 s after ramp pacing. ‘AF maintenance duration’ was defined as the pure period of sustained AF, and ‘AF/ atrial tachycardia (AT) maintenance duration’ indicated an AF or AT sustaining period between 20 s and 300 s. ‘AF defragmentation’ indicated that AF changed to AT or was terminated.

As shown in Fig 1, we measured the spatiotemporal stability of DF, number of PS per 1 ms, and longest PS life-span from 9 AF segment periods (T1-T9: 6-s at every 30-s during AF maintenance) within 300 s (Fig 1A) in 10 segmental regions of the LA (septum, anterior wall, LA appendage, peri-mitral area, posterior inferior wall, posterior wall, left upper and lower pulmonary veins (PVs), and right upper and lower PVs, Fig 1B). To determine the DF, the power spectral density was obtained by Fourier transform of each cell’s virtual action potential at each node, and the DF was defined as the frequency with the highest power. We calculated DF numbers from each of 440,000~500,000 nodes that generate AF action potentials. The highest 10, 15, or 20% DF areas were defined by the nodes that showed top 10%, 15%, or 20% DF numbers [14]. We virtually ablated nodes that showed the highest 10%, 15%, and 20% DF by non-conducted state [7]. Therefore, we reduced 10~20% of critical mass of LA by virtual ablation in this study, and critical mass contribute to the maintenance mechanism of fibrillation [26, 27]. To obtain the spatial and the temporal mean variance, ‘regional proportions of the highest 10% DF area (total 90 values of each patient)’ was calculated during 9 time periods (T1-T9) for each 10 LA segmental regions. The spatial mean variance is the variance of ‘regional proportions of the highest 10% DF area’ with respect to 10 segmental regions at each time period. The temporal mean variance is the variance of ‘regional proportions of the highest 10% DF area’ with respect to 9 time periods (T1-T9) at each LA segmental region.

Phase ‘θ’ at each site (x,y) was calculated as

$$\theta \left(x,y,t\right)=arctan\left[V\left(t+\tau \right)-V_{mean},\;V\left(t\right)-V_{mean}\right],$$ (2)

where the function arctan calculated the phase difference between membrane potential at time t (V(t)) and delayed transmembrane potential at time t+τ (V(t+τ)). At each site (x,y), we calculated V_mean_ (x,y) by averaging the action potential during the whole fibrillation state [28]. V_mean_ plays the role of origin point in the phase space V(t) and V(t+τ) [29, 30]. PS was defined as the point where phase was undetermined [6]. Number of PS was determined by counting the generated PS points per ms. A single PS was defined from when the PS occurred to when the PS disappeared during 6-second PS analysis period. Based on previous studies, we sampled 6-second data for a single PS calculation [6, 14]; still, it was continuous PS monitoring by overlapping consecutive time intervals. Using continuous PS calculation, we generated PS trajectories and calculated PS life span. The points where the PS occurs could be multiple at the same time. Thus, number of PS per 1ms is suitable for expressing all the PS appearing simultaneously. The PS life-span is the time of PS trajectory which is determined by the trace generated by the PS on the atrial surface. The longest PS life-span was defined as one of the longest time length of PS trajectory on the atrial surface.

### Virtual ablation for high DF areas depending on the CV

For virtual DF ablations, the conduction block was applied by adjusting the diffusion coefficient. The ablated region was set to the non-conduction condition to block the electrical conduction. The DF ablations were targeted to the areas with the 10%, 15%, and 20% highest DF, and were conducted at the end of each DF analysis period (T1-T9) with each different CV. AF termination and defragmentation (AF changing to AT) rates were determined within 30 s after the virtual intervention.

### Statistical analysis

Data are expressed as means ± standard deviation. The mean variance was used to test the temporal and spatial stability of DF distribution. Data comparisons were analyzed by paired t-tests. All statistical analyses were performed using SPSS version 19.0 (IBM Corporation, Somers, NY). A p-value of <0.05 was considered statistically significant, and all p-values were compared with CV 0.4 m/s.

## Results

### AF induction and maintenance depending on CV

The patient characteristics are summarized in Table 1. All 10 patients had persistent AF, and 80% of them were male. The mean patient age was 61.8±13.5 years old, and the mean anterior posterior diameter of the left atrium was 48.4±7.9 mm. Most of the patients had normal left ventricular function (ejection fraction 59.2±11.8%). After 4,560ms virtual ramp pacing (200–180 ms), successful AF induction rates were 90%, 90%, 100%, 40%, and 20% at CV 0.2, 0.3, 0.4, 0.5, and 0.6 m/s, respectively (Table 2). AF maintenance duration was the longest in the CV 0.4 m/s condition, but shortest in the CV 0.6 m/s condition (p<0.001, Table 2). Among 34 episodes of successfully induced AF (lasting >20 s after pacing), the spontaneous AF/AT termination rate (within 300 s) was 0% at CV 0.4 m/s, but 100% at CV 0.5m/s (p<0.001) and CV 0.6m/s (p = 0.015, Table 2). The AF defragmentation rate was 0% at CV 0.4 m/s, but 100% at CV 0.5 m/s (p<0.001) and CV 0.6 m/s (p = 0.015, Table 2).

**Table 1:** Patient characteristics.

Age, years (Mean ± SD)	61.8 ± 13.5
> 75 years old	2 (20%)
65–75 years old	1 (10%)
< 65 years old	7 (70%)
Gender	
Male	8 (80%)
Female	2 (20%)
Persistent AF	10 (100%)
Heart failure	0 (0%)
Hypertension	2 (20%)
Diabetes	3 (30%)
Previous stroke	2 (20%)
Previous TIA *	0 (0%)
Vascular disease	3 (30%)
Left atrium dimension	48.4 ± 7.9 mm
Ejection fraction	59.2 ± 11.8%
E/Em **	11.5 ± 6.1

* TIA, transient ischemic attack;

** E/Em, the ratio of early diastolic mitral inflow velocity (E) to early diastolic mitral annular velocity (Em).

**Table 2:** AF maintenance duration for each conduction velocity in the 10 patients.

Conduction Velocity		0.2 m/s	0.3 m/s	0.4 m/s	0.5 m/s	0.6 m/s
Patients		AF maintenance duration (AF/AT maintenance duration)				
A		223s (>300s)	>300s (>300s)	>300s (>300s)	48s (48s)	27s (27s)
B		>300s (>300s)	207s (207s)	>300s (>300s)	5.8s (5.8s)	6.4s (6.4s)
C		>300s (>300s)	211s (211s)	>300s (>300s)	19s (19s)	6.8s (6.8s)
D		35s (>300s)	>300s (>300s)	>300s (>300s)	0s (0s)	0s (0s)
E		>300s (>300s)	11s (>300s)	>300s (>300s)	0s (0s)	0s (0s)
F		0s (0s)	193s (>300s)	>300s (>300s)	93s (93s)	16s (16s)
G		174s (>300s)	22s (>300s)	>300s (>300s)	30s (30s)	0s (0s)
H		218s (>300s)	231s (>300s)	>300s (>300s)	0s (0s)	0s (0s)
I		>300s (>300s)	>300s (>300s)	>300s (>300s)	10.5s (10.5s)	21s (21s)
J		37s (>300s)	118s (>300s)	>300s (>300s)	28s (28s)	14s (14s)
Overall patients	Successful AF induction	90% (9/10)	90% (9/10)	100% (10/10)	40% (4/10)‡	20% (2/10)*
	AF maintenance duration (s)	188.7±115.8‡	189.3±102.1†	>300	23.4±27.7*	9.1±9.4*
Among patients with successfully induced AF	AF/AT termination	0% (0/9)	22.2% (2/9)	0% (0/10)	100% (4/4)*	100% (2/2)‡
	AF/AT maintenance duration (s)	>300	281.8±36.4	>300	49.8±26.2*	24±3*
	AF defragmentation	55.6% (5/9)‡	70% (7/9)*	0% (0/10)	100% (4/4)*	100% (2/2)‡
	AF maintenance duration (s)	209.7±102.5‡	209.1±87.5†	>300	49.8±26.2*	24±3*

*, p<0.001 vs. CV 0.4 m/s;

^†^, p<0.01 vs. CV 0.4 m/s;

^‡^, p<0.05 vs. CV 0.4 m/s;

All p-values vs. CV 0.4m/s

Successful AF induction: Maintaining AF for longer than 20 s

AF maintenance duration: Pure period of sustained AF

AF/AT termination: Terminated AF or AT between 20 s and 300 s

AF/AT maintenance duration: Sustaining period of AF or AT between 20 s and 300 s

AF defragmentation: AF termination or changing to AT

### Spatiotemporal variability of the DF area and PS depending on CV

**Fig 2:**
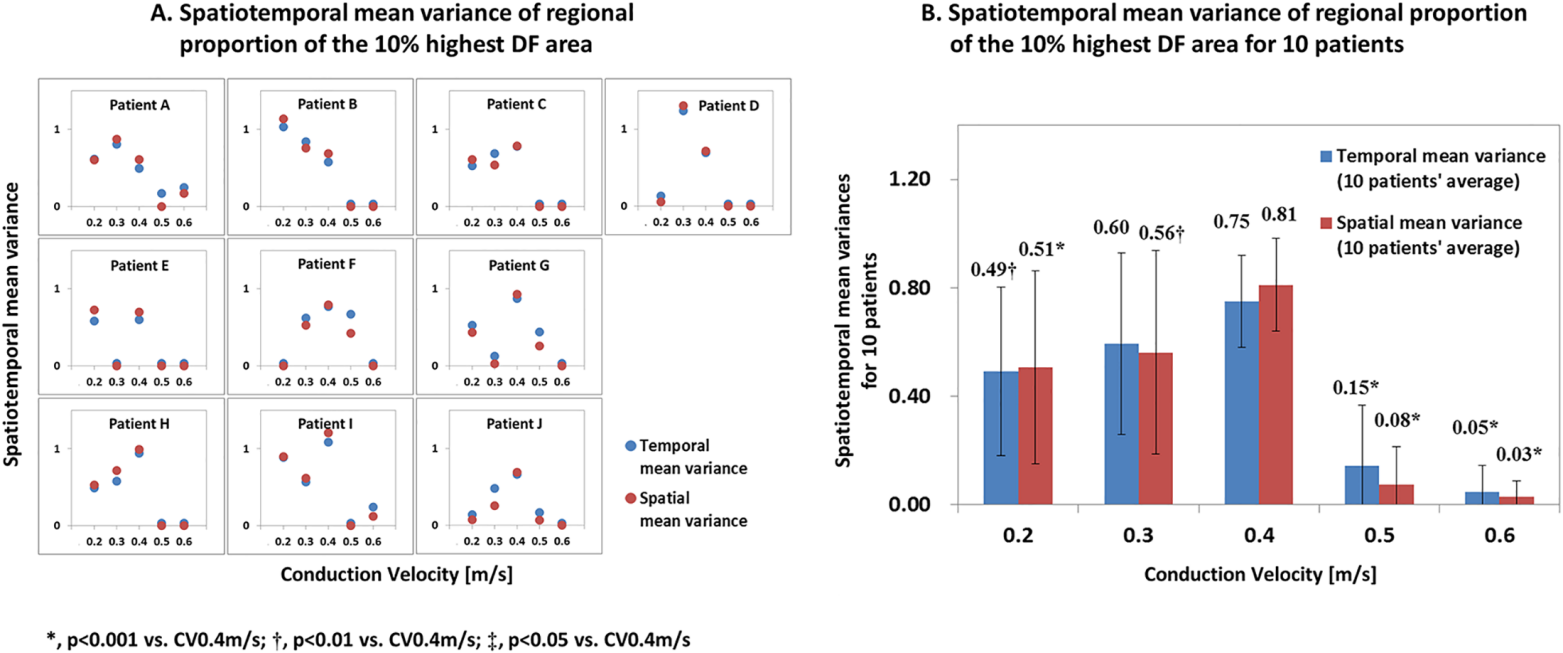
**A.** Spatiotemporal mean variance of regional proportion of the 10% highest DF area. **B.** Spatiotemporal mean variance of regional proportion of the 10% highest DF area in 10 patients.

**Fig 3:**
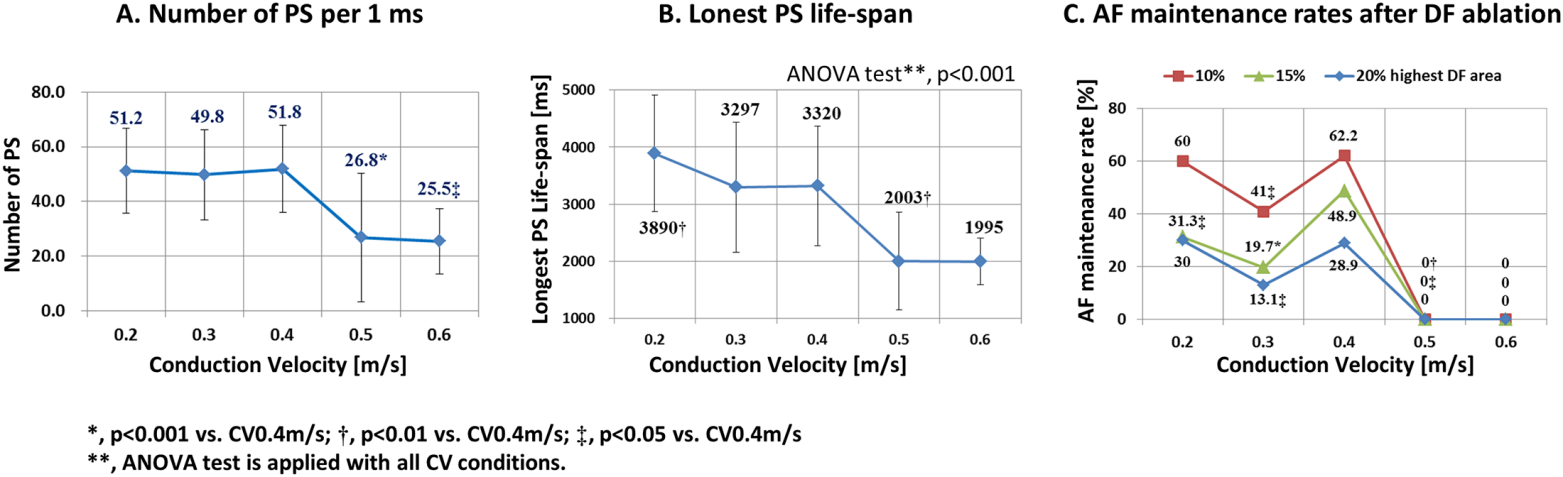
**A.** Number of PS per 1 ms. **B.** The longest PS life-span. **C.** AF maintenance rates after DF ablation.

We calculated DF spatial mean variances (mean variance [SD^2^/(n-1)] of the highest 10% DF proportions in each of the 10 LA regional segments) and temporal mean variances of DF (mean variance of the highest 10% DF proportions in each of the 9 segment periods; T1-T9, Fig 1) at CV 0.2–0.6 m/s conditions (Fig 2A). The spatial mean variance of DF was significantly higher at CV 0.4 m/s than in the other conditions (Fig 2B), and the temporal mean variance of DF was higher at CV 0.4 m/s than CV 0.2 m/s (p = 0.002), CV 0.5 m/s (p<0.001), and CV 0.6 m/s (p<0.001, Fig 2B). The number of PS per ms was significantly higher at CV 0.4 m/s than CV 0.5 m/s (p<0.001) or CV 0.6 m/s (p = 0.023, Fig 3A). The longest PS life-span was significantly higher in conditions with a lower CV (ANOVA, p<0.001, Fig 3B). Under the condition of CV 0.6 m/s, AF easily terminated spontaneously with a low number of PS and shorter PS life-span. Although CV restitution may affect local CV in anisotropic conduction model, both CV restitution and APD restitution were not significantly different depending on CV conditions in this homogeneous monolayer model (S1 Fig).

### Different response to virtual ablation for DF area depending on CV

**Fig 4:**
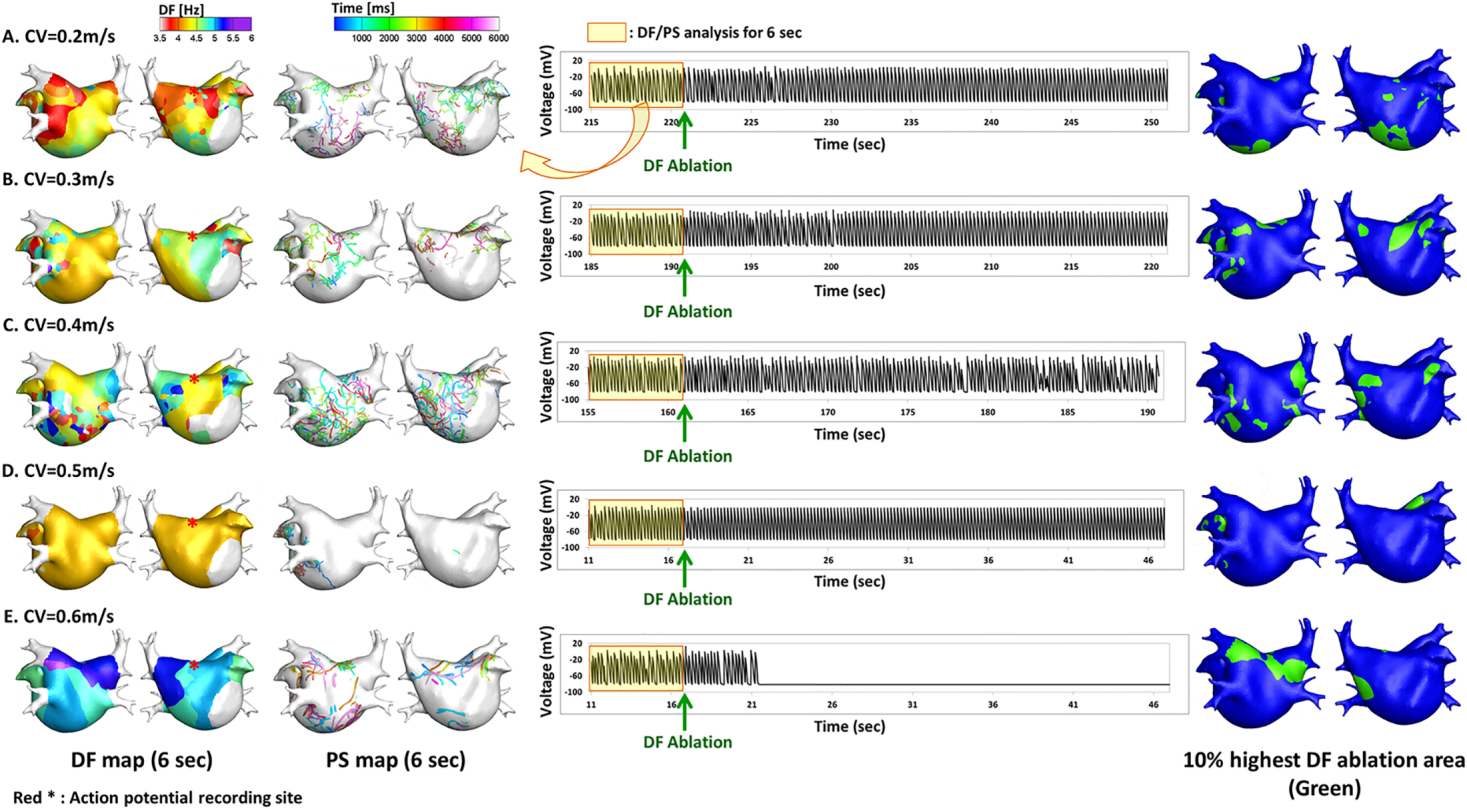
**Left side panels,** Examples of DF and PS maps depending on CV; **Middle panels,** action potential tracings acquired from the high septum of the LA (red asterisk); **Right side panels,** Maps of the highest 10% DF site maps (green areas) depending on each CV, (A) CV = 0.2m/s, (B) CV = 0.3m/s, (C) CV = 0.4m/s, (D) CV = 0.5m/s, and (E) CV = 0.6m/s.

We conducted virtual ablation of DF targeting the highest 10% DF, 15% DF, and 20% DF at the end of each time segment (T1-T9, Fig 1A). After excluding the episodes of spontaneous AF termination or defragmentation (AF changing to AT), there were 60, 61, 90, 7, and 2 episodes virtual DF ablation available at CV 0.2 m/s, 0.3 m/s, 0.4 m/s, 0.5 m/s, and 0.6 m/s conditions, respectively (Table 3). After virtual DF ablation, AF was maintained, defragmented to AT, or terminated depending on CV conditions (Fig 4). Table 3 summarizes the AF termination and AF defragmentation rates after virtual DF ablation targeting the 10%, 15%, and 20% highest DF areas. Despite the limited number of virtual DF ablation available episodes at CV 0.5 m/s and 0.6 m/s conditions (9 episodes), the post-DF ablation AF termination or defragmentation rates were significantly higher at the conditions of CV 0.5 m/s and 0.6 m/s than conditions with lower CVs (Table 3). Fig 3C summarizes the failure rates of AF termination or defragmentation after DF ablation at each CV condition. Failure rates of virtual DF ablation show bimodal patterns similar to the spatiotemporal mean variance of DF, amount of PS per unit time, or the longest PS life-span.

**Table 3:** Outcomes of virtual ablation for high DF area depending on CV.

Conduction Velocity		0.2 m/s	0.3 m/s	0.4 m/s	0.5 m/s	0.6 m/s	ANOVA
**10% DF** **ablation area**	Number of episodes	60	61	90	7	2	
	AF termination	0(0%)	1(1.6%)	1(1.1%)	0(0%)	1(50.0%)‡	p<0.001
	AF defragmentation	24(40.0%)	36(59.0%)‡	34(37.8%)	7(100%)†	2(100%)	p = 0.001
**15% DF** **ablation area**	Number of episodes	60	61	90	7	2	
	AF termination	1(1.7%)	6(9.8%)‡	1(1.1%)	2(28.6%)‡	0(0%)	p = 0.002
	AF defragmentation	41(68.3%)‡	49(80.3%)*	46(51.1%)	7(100%)‡	2(100%)	p<0.001
**20% DF** **ablation area**	Number of episodes	60	61	90	7	2	
	AF termination	2(3.3%)	5(8.2%)	4(4.4%)	1(14.3%)	1(50.0%)	p = 0.048
	AF defragmentation	42(70.0%)	53(86.9%)‡	64(71.1%)	7(100%)	2 (100%)	p = 0.055

*, p<0.001 vs. CV 0.4 m/s;

^†^, p<0.01 vs. CV 0.4 m/s;

^‡^, p<0.05 vs. CV 0.4 m/s;

All p-values vs. CV 0.4m/s

AF defragmentation: AF termination or changing to AT

We compared episodes with AF termination or defragmentation after 10% highest DF ablation to those with failed defragmentation, regardless of CV conditions. The baseline AF maintenance duration was shorter (243.7±81.6 vs. 276.6±50.0 s, p<0.001), spatiotemporal mean variance of DF was lower (0.71±0.51 vs. 0.89±0.57, p = 0.014), and the number of PS per ms was lower (46.6±17.1 vs. 53.1±15.9, p = 0.004) in the episodes of AF termination or defragmentation after virtual DF ablation than in those with failed AF defragmentation.

## Discussion

### Main findings

In this study, we generated 5 different CV conditions, and evaluated the spatiotemporal stability of DF with patient-specific 3D *in-silico* LA modeling. Based on the results, the spatiotemporal stability of the high DF sites varied as the CV changed. The CV affected the variation of the high DF distribution. Virtual DF ablation of the high DF area was more likely to result in defragmented AF under easily terminating AF conditions, but not under long-lasting and sustained AF conditions.

### Controversy in AF rotor ablation

After Konings et al. initially observed complex fractionated atrial electrograms at the areas of slow conduction and/or pivotal points where AF wavelets turn around at the end of the functional blocks, Nademanee et al. reported that this complex fractionated electrogram might be an ideal target site for extensive catheter ablation for persistent AF, reporting a 91% success rate at 1-year follow-up. Eight years after Nademanee’s report, Narayan et al. mapped and visualized the rotor of AF using a multi-electrode basket catheter, and ablation of the rotor terminated or slowed AF and improved rhythm outcome [5]. Haissaguerre et al. also reported that catheter ablation targeting the AF driver domain detected by biatrial geometry relative to an array mapping 252 body surface electrodes significantly reduced ablation time for AF termination. Localized rotors and focal sources have been considered as the prevalent mechanisms which drive and maintain AF for the past few decades [5]. However, focal impulse and rotor mapping (FIRM) and ablation showed inconsistent AF ablation outcome [31], and seems to results in better clinical outcomes in repeat ablation cases [5]. In contrast, high density unipolar mapping [32] or non-invasive electrocardiographic imaging (ECGI) [33] detected very few or no rotors in human AF mapping.

### DF: A rotor of AF or meandering target?

There are several different ways to localize AF rotors, such as PS, DF, or Shannon entropy. Hwang et al. compared the parameters representing a rotor of AF, and the highest DF site was the best parameter to reflect a stable rotor in experimental mapping studies of ventricular fibrillation [34] and an *in-silico* human AF modeling study [7]. Virtual ablation targeting the highest DF coincided with a stable rotor, and virtual ablation targeting the highest DF was effective in AF defragmentation [7]. DF areas have been proven to be the location of the rotor in experimental studies of AF [9]. However, the RADAR-AF clinical trial unfortunately failed to prove the superiority of DF source ablation outcomes compared to conventional ablation [16]. Sequential data point acquisition may raise concerns regarding DF stability, and it is difficult to use electrogram data to localize DF in human AF as it does not natively have the sinusoidal appearance of optically mapped data. Moreover, focal areas of high DF are highly variable spatiotemporally [14, 35]. Even though elimination of a high DF area might be effective in AF termination or defragmentation [7]. DF sites cannot be targeted in conditions with spatiotemporally unstable highly meandering rotors [14, 35]. In the CONFIRM trial, which showed good clinical outcomes of AF rotor ablation, the proportion of prior conventional ablation was 42% [5], and FIRM ablation was proven to be effective in repeat ablation cases [36]. In this study, we proved that the effect of DF ablation differs under different conditions of CV in human AF modeling.

### Conduction velocity and spatiotemporal stability of DF

Spatiotemporal stability of a rotor might be a major determinant for successful or failed DF ablation of AF [7]. The probability of reentry is affected by wavelength, which is proportional to the refractory period and CV [12, 37], critical mass [27], or restitution, which is the dynamic heterogeneity of the refractory period (APD restitution) [38, 39] or CV (CV restitution) [40, 41]. It has been reported that fibrillation patterns can change from meandering multiple wavelets to a focal source and stable rotor depending on tissue excitability of CV in *ex-vivo* ventricular tissue [42]. Therefore, we tested rotor mapping and ablation at several different CV conditions in an *in-silico* human AF model. The advantage of computer simulation modeling is the ability to repeat tests under various conditions precisely and reproducibly, which cannot be done in experimental or clinical settings. The current study showed bimodal trend of AF wave-dynamics depending on CVs. At high CVs, long wavelengths with a few peripheral wave-breaks and a small number of PS allowed AF to easily terminate with or without DF ablation. In low CV conditions, AF was easily inducible, well-maintained, and difficult to terminate due to continuous wave-breaks with short wavelengths and high number of PSs. However, organized reentries were well-maintained with poor wave-breaks at very low CV conditions despite the short wavelength, similar to slow and very organized AF in patients with rheumatic valvular disease with severe structural remodeling of atria. Those organized wavelets at very low CV with large core size can be terminated by colliding to the anatomical boundaries that show biphasic pattern of DF ablation outcome, depending on CVs. There have been several studies demonstrating relatively spatiotemporally stable DF under clinical conditions [10]. In contrast, other studies reported differences in the DF distribution in patients with paroxysmal and persistent AF [10, 35]. Therefore, we need to recognize the limitations of low resolution sequential acquisition of clinical bipolar mapping and variable rotor conditions depending on CV, refractory period, or critical mass. High DF areas may posit rotors or drivers of AF [9, 10], and ablation of high DF areas terminates or defragments AF [10]. However, the spatiotemporal stability of the DF area is highly dependent on electrophysiological conditions including CV, and it is difficult to target the DF area for ablation due to unstable meandering. Under conditions with low AF inducibility and maintenance rate, AF is vulnerable to termination after DF ablation. Therefore, whether DF ablation terminates AF or if easily terminating AF shows a stable rotor remains to be clarified. Fibrillation wavelets drift to align their axis of rotation, filaments, with the minimal resistance line which approximately aligns with the myocardial fibers [43]. However, a rotor still meanders under homogeneous distribution of ionic properties, whereas it drifts under heterogeneous distribution of ionic properties [44]. In this study, we focused on the physiologic effects of CV on rotor or DF, because constant local CV can be defined only under homogeneous model. But, our results were reproducible in the simplified 2D modeling which incorporated fiber orientation (S2 Fig). Although rotors tend to anchor at boundaries of different wall thicknesses [45], or sites with different transmural fiber orientation [46], and increased fibrosis [47], we tried to explore specific effects of CV on spatiotemporal stability of rotors.

### Limitation

There are some limitations of this study. The geometry of the LA is patient-specific. However, this study used a structurally homogeneous LA model, which did not include individual electrophysiological and structural characteristics such as regional differences in action potential shape, fiber orientation, focal fibrosis [21], and LA wall thickness. However, wave dynamics in the monolayer model were reported to be similar to that in a bilayer model except for the area of abrupt change of fiber orientation [48]. Anisotropic fiber orientations in atrial tissue contribute to the initiation and maintenance mechanisms of reentry [49, 50]. Although homogeneous model utilized in this study has a significant limitation as an isotropic conduction model, it is still useful to explain the mechanisms of rotor ablations for the following reasons. First, the contribution of anisotropy to reentry is mostly PV fibers around PV in the previous studies [51], but PV isolation is performed without exception in clinical catheter ablation. Second, Polontchouk et al. [52] reported that the anisotropy effect was not significant in the pathophysiological state due to AF-induced gap junction remodeling. Third, our previous patient-specific simulation studies showed similarity of clinically acquired AF map (CFAE map) and isotropic AF modeling map [53, 54]. Further, ionic current conditions were spatially uniform in the model, and spatial heterogeneity in the ionic current properties would have affected the wave dynamics. As the regions of the LA were divided manually based on a clinical ablation strategy, the size and shape of each LA region was not uniform. Real atrium has heterogeneous structures and pacing near the Buchman’s bundle can result in different conduction patterns, given the anisotropic conduction at this region. MRI imaging based human atrial anatomical model (e.g. Gong et al., Circulation 2007), which includes realistic diffusion tensor data, may overcome this limitation [55].

## Conclusion

The CV has an effect on the distribution of the high DF area. By changing the CV, the spatiotemporal variability of the high DF area changed. DF ablation was more likely to terminate or defragment AF with spatiotemporally stable DF, but not likely to do so in long-lasting and sustained AF conditions, depending on CV.

## Supporting information

S1 Fig **A.** CV restitution curves and **B-F.** APD restitution curves depending on preset CVs. Restitution curves were generated by dynamic ramp pacing protocol (pacing cycle length of 500 ms~196 ms excluding induced AF data. We failed 1:1 capture from pacing cycle length of 210ms in CV 0.2m/s, 208ms in CV 0.3m/s, and 202ms in CV0.4m/s, respectively.).
(TIF)

S2 Fig 2D modeling at each CV (0.2~0.6m/s) incorporated with fiber orientation. **A.** DF and PS map at each CV. The fiber orientation is implemented as transversal and longitudinal conduction ratio 1:2. A 600 x 600 element cell array was used to simulate. Spatial discretization is 0.25mm and temporal discretization is 0.1ms. In low CV conditions, AF was well maintained and difficult to terminate due to continuous wavebreaks and short wavelength. Organized reentries were observed in CV 0.2m/s. In high CV conditions, AF was easily terminated than in low CV due to long wavelengths and few peripheral wavebreaks. Average DF value is significantly lower at CV 0.2m/s (6.35±0.10Hz) and 0.3m/s (6.19±0.13Hz) than CV 0.4m/s (p<0.001) and significantly higher at CV 0.6m/s (6.58±0.03Hz) than CV 0.4m/s (p<0.001). Number of PS is the highest (20348) at CV 0.2m/s and the lowest (2957) at CV 0.6m/s. AF maintenance duration becomes longer as the CV becomes smaller. **B.** Spatiotemporal mean variance of regional proportion of the 10% highest DF area. Spatiotemporal stability of DF was analyzed from 9 segmental regions and 3 time periods. **C.** Number of PS. **D.** AF maintenance rates after DF ablation.
(TIF)
