# Supplementary figures and images for: Effectiveness of atrial fibrillation rotor ablation is dependent on conduction velocity: An in-silico 3-dimensional modeling study
Source: PLoS One. 2017 Dec 29;12(12):e0190398. doi: 10.1371/journal.pone.0190398 (PMC5747478; doi:10.1371/journal.pone.0190398)

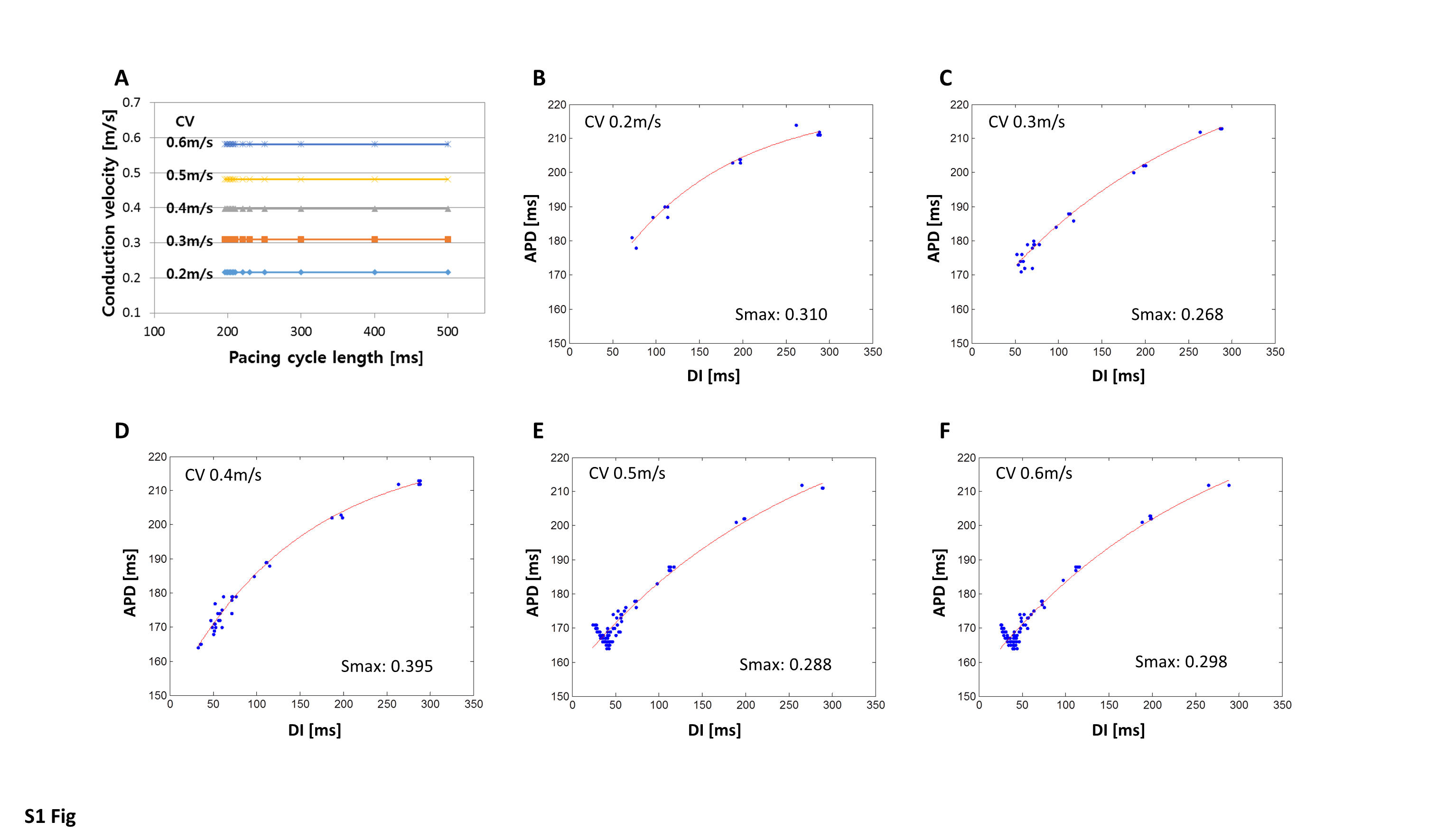

Supplement: S1 Fig — A. CV restitution curves and B-F. APD restitution curves depending on preset CVs. Restitution curves were generated by dynamic ramp pacing protocol (pacing cycle length of 500 ms~196 ms excluding induced AF data. We failed 1:1 capture from pacing cycle length of 210ms in CV 0.2m/s, 208ms in CV 0.3m/s, and 202ms in CV0.4m/s, respectively.). (TIF) [file pone.0190398.s001.TIF]

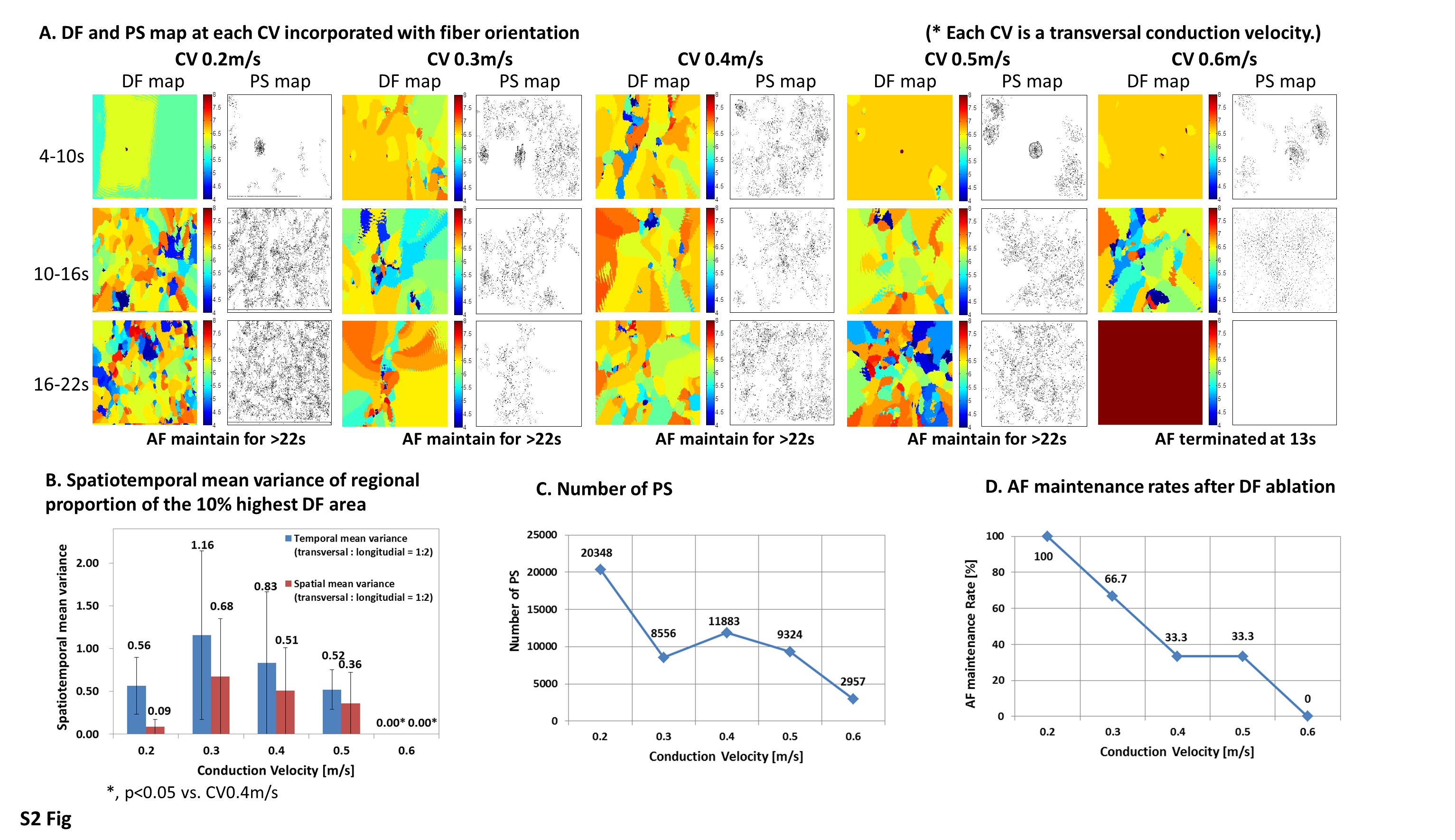

Supplement: S2 Fig — A. DF and PS map at each CV. The fiber orientation is implemented as transversal and longitudinal conduction ratio 1:2. A 600 x 600 element cell array was used to simulate. Spatial discretization is 0.25mm and temporal discretization is 0.1ms. In low CV conditions, AF was well maintained and difficult to terminate due to continuous wavebreaks and short wavelength. Organized reentries were observed in CV 0.2m/s. In high CV conditions, AF was easily terminated than in low CV due to long wavelengths and few peripheral wavebreaks. Average DF value is significantly lower at CV 0.2m/s (6.35±0.10Hz) and 0.3m/s (6.19±0.13Hz) than CV 0.4m/s (p<0.001) and significantly higher at CV 0.6m/s (6.58±0.03Hz) than CV 0.4m/s (p<0.001). Number of PS is the highest (20348) at CV 0.2m/s and the lowest (2957) at CV 0.6m/s. AF maintenance duration becomes longer as the CV becomes smaller. B. Spatiotemporal mean variance of regional proportion of the 10% highest DF area. Spatiotemporal stability of DF was analyzed from 9 segmental regions and 3 time periods. C. Number of PS. D. AF maintenance rates after DF ablation. (TIF) [file pone.0190398.s002.tif]
